# Author Correction: Temporal and sex-dependent gene expression patterns in a renal ischemia–reperfusion injury and recovery pig model

**DOI:** 10.1038/s41598-024-58876-0

**Published:** 2024-04-09

**Authors:** Stéphane Nemours, Luis Castro, Didac Ribatallada-Soriano, Maria E. Semidey, Miguel Aranda, Marina Ferrer, Alex Sanchez, Joan Morote, Gerard Cantero-Recasens, Anna Meseguer

**Affiliations:** 1 Renal Physiopathology Group, Vall d’Hebron Research Institute, Passeig Vall d’Hebron 119-129, 08035 Barcelona, Spain; 2 Biomedical Research in Urology Group, Vall d’Hebron Research Institute, Barcelona, Spain; 3 Department of Pathology, Hospital Vall d’Hebron, Barcelona, Spain; 4 Rodent Platform, Vall d’Hebron Research Institute, Universitat Autònoma de Barcelona, Barcelona, Spain; 5 Unitat d’Estadística I Bioinformàtica, (UEB), Vall d’Hebron Research Institute, Barcelona, Spain; 6 Department of Genetics, Microbiology and Statistics, Universitat de Barcelona, Barcelona, Spain; 7 Departament de Bioquímica I Biologia Molecular, Unitat de Bioquímica de Medicina, Universitat Autònoma de Barcelona, Bellaterra, Spain; 8 Red de Investigación Renal (REDINREN), Instituto Carlos III-FEDER, Madrid, Spain

Correction to: *Scientific Reports* 10.1038/s41598-022-10352-3, published online 28 April 2022

The Data Availability section in the original version of this Article was omitted.

It now appears as below:

“All microarray data in this publication have been deposited in NCBI's Gene Expression Omnibus^57,58^ and are accessible through GEO Series accession number GSE259281 (https://www.ncbi.nlm.nih.gov/geo/query/acc.cgi?acc=GSE259281).”

The original Article has been corrected.

