# Supplementary material for: Temporal and sex-dependent gene expression patterns in a renal ischemia–reperfusion injury and recovery pig model
Source: Sci Rep. 2022 Apr 28;12:6926. doi: 10.1038/s41598-022-10352-3 (PMC9051203; doi:10.1038/s41598-022-10352-3)
Supplement: Supplementary file 1 — Supplementary Information. [file 41598_2022_10352_MOESM1_ESM.pdf]

SUPPLEMENTARY INFORMATION

Temporal and sex-dependent gene expression patterns in a renal ischemia-reperfusion injury and recovery pig model

Stéphane Nemours[1], Luis Castro[2], Didac Ribatallada-Soriano[1], Maria Eugenia Semidey[3], Miguel Aranda[1], Marina Ferrer[4], Alex Sanchez[5,6], Joan Morote[2], Gerard Cantero-Recasens[1], Anna Meseguer[1,7,8,#]

Affiliations: [1]Renal Physiopathology Group, CIBBIM-Nanomedicine, Vall d'Hebron Research Institute, Barcelona, Spain. [2]Biomedical Research in Urology Group, Vall d'Hebron Research Institute, Barcelona, Spain. [3]Department of Pathology, Hospital Vall d'Hebron, Barcelona, Spain. [4]Rodent Platform, Vall d'Hebron Research Institute, Universitat Autònoma de Barcelona, Barcelona, Spain. [5]Unitat d'Estadística i Bioinformàtica, (UEB), Vall d'Hebron Research Institute, Barcelona, Spain. [6]Department of Genetics, Microbiology and Statistics. Universitat de Barcelona, Barcelona, Spain, [7]Departament de Bioquímica i Biologia Molecular, Unitat de Bioquímica de Medicina, Universitat Autònoma de Barcelona, Bellaterra; Spain. [8]Red de Investigación Renal (REDINREN), Instituto Carlos III-FEDER, Madrid, Spain.

# Correspondence to:
Anna Meseguer: ana.meseguer@vhir.org
Renal Physiopathology Group, CIBBIM-Nanomedicine, Vall d'Hebron Research Institute,
Passeig Vall d'Hebron 119-129, 08035 Barcelona, Spain.
Telephone number: +34 934894070; fax number: +34 934894015;

INDEX

Supplementary figures

Supplementary figure 1 .... Page 3
Supplementary figure 2 .... Page 4
Supplementary figure 3 .... Page 5

Supplementary tables

Supplementary table 1 .... Page 6
Supplementary table 2 .... Page 7
Supplementary table 3 .... Page 8
Supplementary table 4 .... Page 9
Supplementary table 5 .... Page 10
Supplementary table 6 .... Page 11
Supplementary table 7 .... Page 12
Supplementary table 8 .... Page 13
Supplementary table 9 .... Page 14
Supplementary table 10 .... Page 15
Supplementary table 11 .... Page 16

SUPPLEMENTARY FIGURE 1

A)

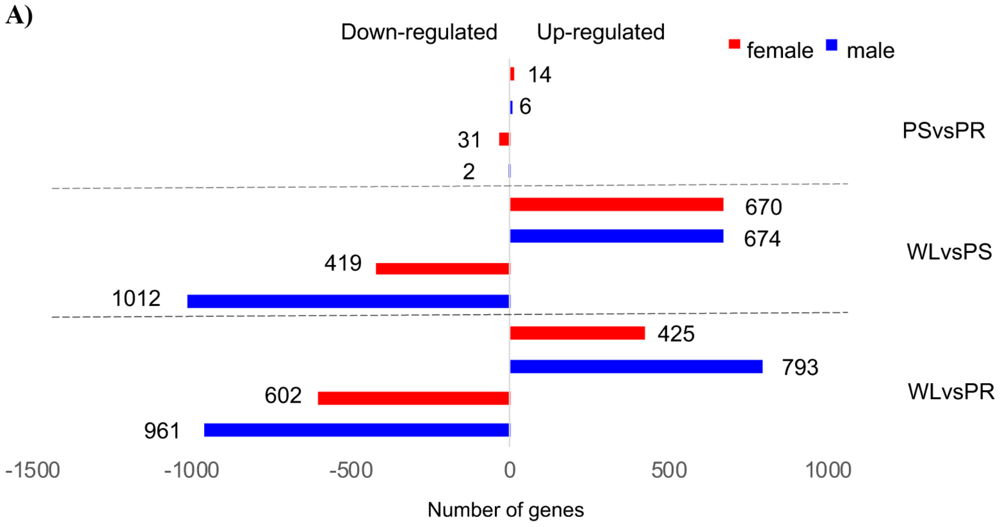

Supplementary figure 1. Number of genes differentially expressed throughout renal IRI in porcine kidney males and females at different time points (adjusted p-value ≤0.25 were considered. (PR: pre-ischemia; PS: post-ischemia; WL: one week later).

SUPPLEMENTARY FIGURE 2

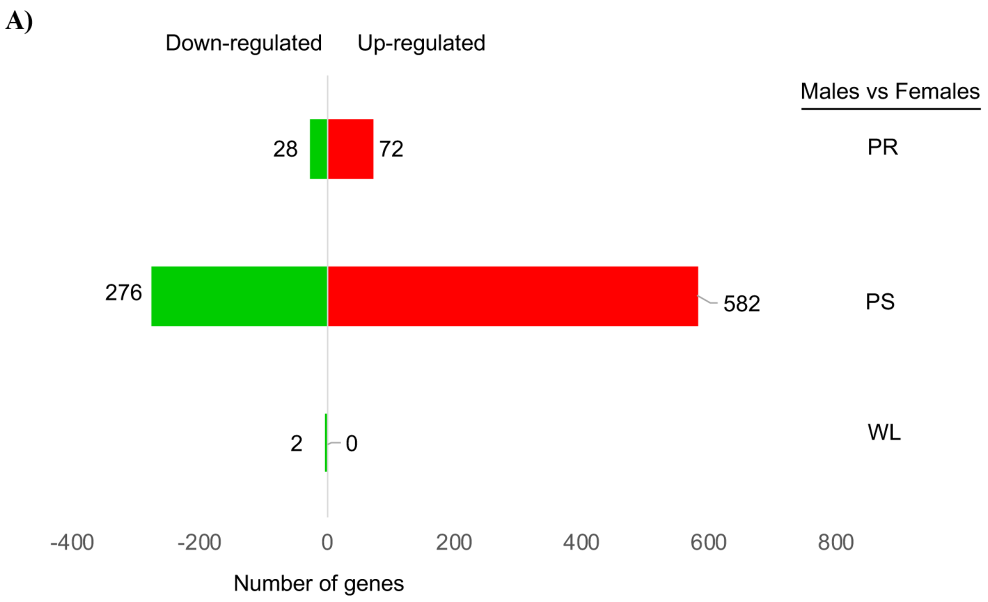

Supplementary figure 2. Number of genes differentially expressed throughout renal IRI in porcine kidney between males and females at the same time point (adjusted p-value ≤0.25 were considered. (PR: pre-ischemia; PS: post-ischemia; WL: one week later).

SUPPLEMENTARY FIGURE 3

A)

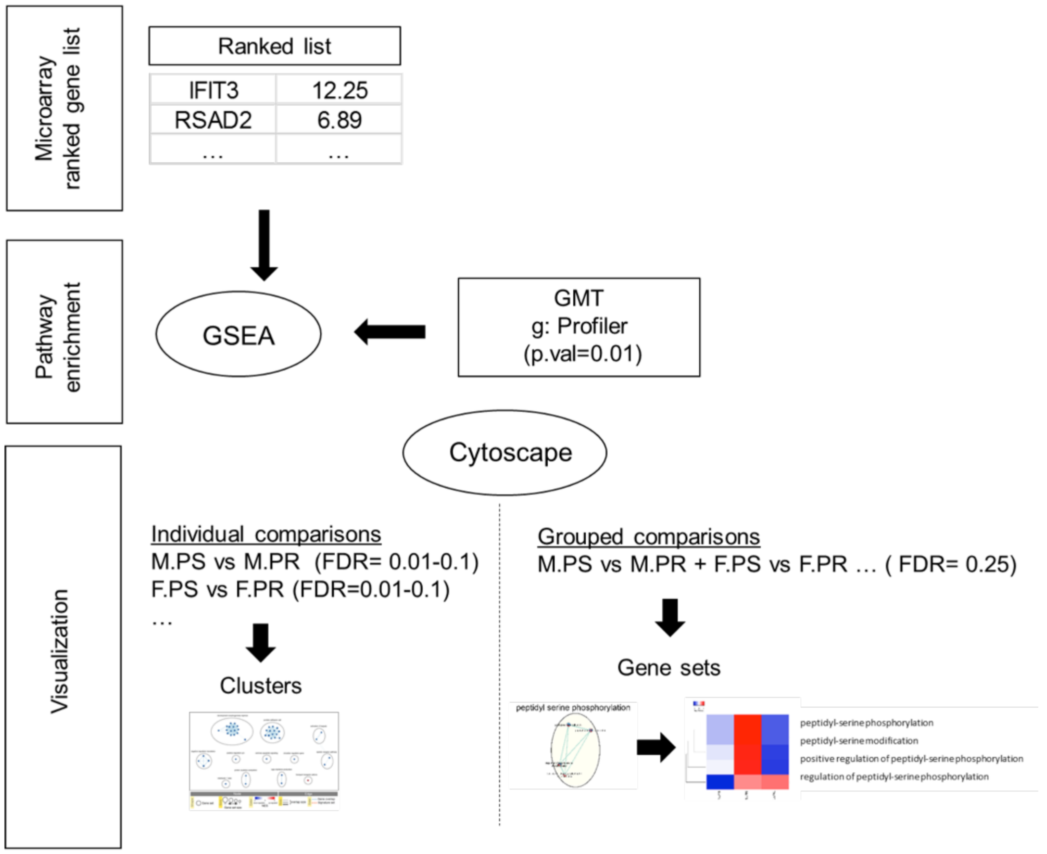

Supplementary figure 3. Protocol overview of GSEA analysis. Gene lists derived from diverse omics data undergo pathway enrichment analysis, using GSEA, to identify pathways that are enriched in the experiment. Pathway enrichment analysis results are visualized and interpreted in Cytoscape using its EnrichmentMap, AutoAnnotate, WordCloud and clusterMaker2 applications.

SUPPLEMENTARY TABLE 1

Time point	SCr (mg/dl)				BUN (mg/dl)			
	Males		Females		Males		Females	
	Individual value	mean	Individual value	mean	Individual value	mean	Individual value	mean
PR	0.79	1.14 ± 0.32	1.01	1.14 ± 0.31	21	18 ± 6	6	15 ± 11
	1.11		1.32		14		25	
	1.24		1.26		24		26	
	1.32		0.82		15		6	
	1.23		1.27		17		13	
PS	1.45	1.69 ± 0.46	1.37	1.71 ± 0.58	31	21 ± 9	8	19 ± 14
	1.67		2.28		16		32	
	1.78		1.91		24		32	
	1.89		1.28		17		7	
	1.65		1.73		18		15	
1d	3.04	2.97 ± 0.86	2.73	3.36 ± 1.17	67	55 ± 16	46	58 ± 22
	2.65		4.46		47		78	
	3.61		3.84		58		70	
	2.7		2.23		47		44	
	2.84		3.53		54		53	
3d	1.75	1.8 ± 0.60	1.6	2.17 ± 0.65	30	27 ± 13	26	35 ± 14
	1.68		2.78		20		51	
	2.28		2.33		41		39	
	1.82		1.55		26		28	
	1.47		2.61		16		33	
WL	1.26	1.62 ± 0.41	1.42	1.79 ± 0.51	19	27 ± 8	22	25 ± 9
	1.84		2.08		35		27	
	1.61		1.83		26		31	
	1.62		-		23		-	
	1.77		1.84		30		21	

Table S1. Serum creatinine and BUN levels in male and female pigs throughout the experiment.

SUPPLEMENTARY TABLE 2

Position number	up-regulated Name	NES	down-regulated Name	NES
1	regulation of transcription from RNA polymerase II promoter in response to stress	1.8810	positive regulation of endothelial cell proliferation	-1.9366
2	regulation of DNA-templated transcription in response to stress	1.8261	regulation of endothelial cell proliferation	-1.9203
3	cellular response to reactive oxygen species	1.8227	regulation of protein processing	-1.9193
4	cellular response to oxidative stress	1.8098	regulation of protein maturation	-1.9115
5	macromolecule methylation	1.6995	Regulation of Complement cascade	-1.8253
6	regulation of anion transport	1.6212	phagocytosis, engulfment	-1.8152
7	protein methylation	1.6147	glial cell differentiation	-1.8119
8	protein alkylation	1.6118	regulation of protein tyrosine kinase activity	-1.8024
9	stem cell population maintenance	1.5914	protein activation cascade	-1.7919
10	maintenance of cell number	1.5812	complement cascade	-1.7897

Table S2. Top 10 up- and down-regulated gene sets and NES in GSEA analysis for FPS vs FPR comparison.

SUPPLEMENTARY TABLE 3

Position number	up-regulated Name	NES	down-regulated Name	NES
1	transport of inorganic cations/anions and amino acids/oligopeptides	2.0897	kidney epithelium development	-2.2511
2	drug transmembrane transport	1.9381	regulation of translation	-2.2027
3	the citric acid (TCA) cycle and respiratory electron transport	1.9151	peptide biosynthetic process	-2.1876
4	complement activation	1.8511	translation	-2.1730
5	protein folding	1.8086	cell junction assembly	-2.1662
6	import across plasma membrane	1.8068	positive regulation of cell-matrix adhesion	-2.1179
7	SLC-mediated transmembrane transport	1.7834	focal adhesion assembly	-2.1149
8	neutrophil activation involved in immune response	1.7833	cell-substrate adherens junction assembly	-2.0967
9	regulation of humoral immune response	1.7830	negative regulation of translation	-2.0765
10	amino acid transmembrane transport	1.7809	adherens junction assembly	-2.0650

Table S3. Top 10 up- and down-regulated gene sets and NES in GSEA analysis for FWL vs FPS comparison.

SUPPLEMENTARY TABLE 4

Position number	up-regulated Name	NES	down-regulated Name	NES
1	amino acid transmembrane transport	2.1240	positive regulation of cell-substrate adhesion	-2.1642
2	transport of inorganic cations/anions and amino acids/oligopeptides	2.0135	regulation of cell-matrix adhesion	-2.0960
3	transition metal ion transport	1.9820	positive regulation of growth	-2.0871
4	carboxylic acid transmembrane transport	1.9330	regulation of cell-substrate adhesion	-2.0625
5	organic acid transmembrane transport	1.9092	positive regulation of cell-matrix adhesion	-2.0569
6	drug transmembrane transport	1.9085	cell-matrix adhesion	-2.0351
7	amino acid transport	1.8928	antigen receptor-mediated signaling pathway	-2.0315
8	electron transport chain	1.8813	cell-substrate adhesion	-2.0033
9	L-amino acid transport	1.8500	rho protein signal transduction	-2.0028
10	SLC-mediated transmembrane transport	1.8089	cell junction organization	-1.9762

Table S4. Top 10 up- and down-regulated gene sets and NES in GSEA analysis for FWL vs FPR comparison.

SUPPLEMENTARY TABLE 5

Position number	up-regulated Name	NES	down-regulated Name	NES
1	positive regulation of neuron death	2.0422	regulation of cell-matrix adhesion	-2.0901
2	regulation of mitotic nuclear division	1.9928	cell-matrix adhesion	-1.9135
3	protein folding	1.9418	focal adhesion assembly	-1.8737
4	cellular responses to external stimuli	1.9348	negative regulation of cell-substrate adhesion	-1.8620
5	protein targeting	1.9206	cell-substrate adherens junction assembly	-1.8569
6	response to topologically incorrect protein	1.8758	adherens junction assembly	-1.8285
7	vacuolar transport	1.8584	cell-substrate junction assembly	-1.8211
8	response to unfolded protein	1.8534	positive regulation of cell-matrix adhesion	-1.7913
9	carboxylic acid transmembrane transport	1.8473	positive regulation of interleukin-6 production	-1.7909
10	coenzyme metabolic process	1.8385	mast cell activation	-1.7906

Table S5. Top 10 up- and down-regulated gene sets and NES in GSEA analysis for MPS vs MPR comparison.

SUPPLEMENTARY TABLE 6

Position number	up-regulated Name	NES	down-regulated Name	NES
1	microtubule cytoskeleton organization	2.2836	defense response to virus	-2.6040
2	mitotic prometaphase	2.0927	defense response to other organism	-2.5883
3	nuclear division	1.9101	response to virus	-2.4996
4	microtubule-based process	1.8978	response to type I interferon	-2.3965
5	outflow tract morphogenesis	1.8861	Interferon Signaling	-2.3915
6	G2/M transition of mitotic cell cycle	1.8435	interferon-gamma-mediated signaling pathway	-2.3862
7	Cilium Assembly	1.8357	cytokine Signaling in Immune system	-2.3738
8	cytokinesis	1.8142	cellular response to type I interferon	-2.3737
9	meiotic cell cycle	1.8006	type I interferon signaling pathway	-2.3710
10	microtubule organizing center organization	1.7707	interferon alpha/beta signaling	-2.3660

Table S6. Top 10 up- and down-regulated gene sets and NES in GSEA analysis for MWL vs MPS comparison.

SUPPLEMENTARY TABLE 7

Position number	up-regulated Name	NES	down-regulated Name	NES
1	microtubule cytoskeleton organization	2.1985	defense response to other organism	-2.7435
2	mitotic prometaphase	2.0865	defense response to virus	-2.6592
3	microtubule organizing center organization	2.0785	response to virus	-2.6044
4	microtubule-based process	2.0765	interferon signaling	-2.5913
5	G2/M transition of mitotic cell cycle	1.9769	interferon alpha/beta signaling	-2.5609
6	nuclear division	1.9130	cytokine signaling in immune system	-2.5028
7	cell cycle, mitotic	1.8798	interferon-gamma-mediated signaling pathway	-2.4957
8	cytokinesis	1.8634	regulation of type I interferon production	-2.4658
9	cell cycle G2/M phase transition	1.8268	innate immune response	-2.4328
10	regulation of microtubule cytoskeleton organization	1.8111	type I interferon production	-2.4251

Table S7. Top 10 up- and down-regulated gene sets and NES in GSEA analysis for MWL vs MPR comparison.

SUPPLEMENTARY TABLE 8

Position number	up-regulated Name	NES	down-regulated Name	NES
1	cytokine signaling in immune system	3.1556	ventricular septum development	-1.8453
2	defense response to other organism	3.1034	keratinocyte differentiation	-1.7788
3	defense response to virus	3.0129	cardiac septum morphogenesis	-1.7693
4	innate immune response	3.0062	cardiac septum development	-1.7458
5	Interferon Signaling	3.0007	outflow tract morphogenesis	-1.7246
6	response to virus	2.9072	glomerulus development	-1.7167
7	cytokine-mediated signaling pathway	2.8606	cardiac ventricle development	-1.6914
8	cellular response to type I interferon	2.8605	detection of stimulus	-1.6709
9	type I interferon signaling pathway	2.8415	brain development	-1.6628
10	response to biotic stimulus	2.8282	regulation of synapse organization	-1.6598

Table S8. Top 10 up- and down-regulated gene sets and NES in GSEA analysis for MPR vs FPR comparison.

SUPPLEMENTARY TABLE 9

Position number	up-regulated Name	NES	down-regulated Name	NES
1	innate immune response	2.6989	defense response to bacterium	1.7816
2	response to virus	2.6627	signal transduction by protein phosphorylation	1.7820
3	Interferon Signaling	2.6063	antigen receptor-mediated signaling pathway	1.7830
4	defense response to virus	2.5981	leukocyte migration	1.7831
5	cytokine signaling in immune system	2.5912	immune response-regulating cell surface receptor signaling pathway	1.7876
6	defense response to other organism	2.5810	zymogen activation	1.7897
7	cytokine-mediated signaling pathway	2.5280	alpha-beta T cell differentiation	1.7904
8	response to interferon-gamma	2.4697	negative regulation of phosphate metabolic process	1.7904
9	type I interferon signaling pathway	2.4601	negative regulation of phosphorus metabolic process	1.7922
10	Interferon gamma signaling	2.4419	regulation of protein localization	1.7928

Table S9. Top 10 up- and down-regulated gene sets and NES in GSEA analysis for MPS vs FPS comparison.

SUPPLEMENTARY TABLE 10

Position number	up-regulated Name	NES	down-regulated Name	NES
1	antigen receptor-mediated signaling pathway	2.5851	carboxylic acid catabolic process	-2.3959
2	immune response-activating cell surface receptor signaling pathway	2.5808	organic acid catabolic process	-2.3600
3	B cell activation	2.3594	cellular amino acid metabolic process	-2.2771
4	B cell proliferation	2.3559	SLC-mediated transmembrane transport	-2.2678
5	Signaling by Interleukins	2.3212	oxidation-reduction process	-2.2151
6	rho GTPase cycle	2.2947	cellular amino acid catabolic process	-2.2092
7	regulation of small GTPase mediated signal transduction	2.2760	alpha-amino acid metabolic process	-2.2010
8	positive regulation of cell cycle process	2.2602	monocarboxylic acid catabolic process	-2.1616
9	microtubule cytoskeleton organization	2.2306	sodium ion transport	-2.1371
10	T cell receptor signaling pathway	2.2300	fatty acid metabolic process	-2.1338

Table S10. Top 10 up- and down-regulated gene sets and NES in GSEA analysis for MWL vs FWL comparison.

SUPPLEMENTARY TABLE 11

Sample	Sex	Age (yrs)	Tumor type	Cardiovascular pathology	Renal pathology	Diabetes	Others
1	M	36	ccRCC	n.a.	n.a.	n.a.	n.a.
2	M	40	ccRCC	Dyslipidemia		NO	Glaucoma
3	M	56	ccRCC			NO	Leukocytic vasculitis
4	M	66	Lung cancer*	n.a.	n.a.	n.a.	n.a.
5	M	69	CRCC	n.a.	n.a.	n.a.	n.a.
6	M	70	ccRCC	n.a.	n.a.	n.a.	n.a.
7	M	70	ccRCC	Atrial fibrillation, heart failure, stroke	CKD	NO	Former smoker
8	M	76	ccRCC	n.a.	n.a.	n.a.	n.a.
9	M	80	ccRCC	n.a.	n.a.	n.a.	n.a.
10	M	Unknown	ccRCC/PRCC	Ischemic heart disease, arterial hypertension	Polycystic kidney disease	NO	Former smoker
11	F	53	ccRCC	n.a.	n.a.	n.a.	n.a.
12	F	53	ccRCC	High blood pressure, dyslipidemia		NO	Goiter
13	F	54	ccRCC	High blood pressure		NO	Goiter, cholecystectomy
14	F	63	ccRCC	n.a.	n.a.	n.a.	n.a.
15	F	65	CRCC	High blood pressure, dyslipidemia, ischemic cardiomyopathy, peripheral artery disease	CKD	NO	Psoriasis
16	F	67	ccRCC	High blood pressure, ischemic stroke		NO	Asthma, brain meningioma
17	F	68	ccRCC	n.a.	n.a.	n.a.	n.a.
18	F	68	ccRCC	n.a.	n.a.	n.a.	n.a.
19	F	83	ccRCC	High blood pressure, atrial fibrillation		NO	Cholecystectomy, hemorrhoidectomy

M: male; F: female; ccRCC: clear cell renal cell carcinoma; CRCC: cromophobe cell carcinoma; PRCC: papillary renal cell carcinoma, CKD: Chronic Kidney Disease, n.a.: not available
*Lung carcinoma with metastase to kidney

Table S11. Patients' clinical data.
